# Effect of Demographic Characteristics and Personality Traits on Eating Patterns in the Context of Dietary Intervention: The EATMED Case Study

**DOI:** 10.3390/ijerph22071095

**Published:** 2025-07-10

**Authors:** Michele Ricci, Andrea Devecchi, Riccardo Migliavada, Maria Piochi, Luisa Torri

**Affiliations:** 1 University of Gastronomic Sciences of Pollenzo, 12042 Bra, CN, Italy; m.ricci@unisg.it (M.R.); r.migliavada@unisg.it (R.M.); m.piochi@unisg.it (M.P.); l.torri@unisg.it (L.T.); 2 Department of Medical Sciences, University of Turin, 10124 Turin, TO, Italy

**Keywords:** Mediterranean diet, digital dietary intervention, attrition analysis, HTAS

## Abstract

There is a confirmed and ongoing need to encourage adherence to healthy dietary patterns in the general population in western societies, given their recognized positive impact in preventing non-communicable diseases (NCDs). A potentially very effective solution is the use of digital tools such as apps and web apps, which can reach a large number of people quickly. Still, to be effective, it is necessary to better understand how participant engagement in these interventions works, to identify the motivations that may lead them to drop out, and to evaluate the effectiveness of these interventions. In our study, an innovative web app designed to encourage adherence to the Mediterranean diet (EATMED) was tested in an intervention study, evaluating adherence to the Mediterranean diet before and after the use of the web app in a cohort of people, compared to a control group, using the MEDI-lite questionnaire. The Health and Taste Attitude Scale questionnaire was also administered to all participants to assess interest in healthy foods and diets, as well as attitudes toward food. The study showed that the score of the Food as Reward subscale of the HTAS had a significant effect on dropout from the intervention study, and that the use of the app among participants who remained resulted in a two-point increase in adherence to the Mediterranean diet, according to the MEDI-lite questionnaire. These results indicate the effectiveness of the EATMED tool and provide useful insights into how to understand and mitigate dropout in digital nutrition interventions.

## 1. Introduction

Unhealthy dietary patterns characterized by a high intake of calories, heavily processed foods, and animal-based foods are rising among the European population [1], increasing the burden of obesity and contributing to environmental degradation [2]. The occurrence of obesity and overweight, defined as excessive adiposity, with or without abnormal distribution or function of the adipose tissue [3], is rising globally, causing harm at both individual and societal levels [4]. Obesity is directly linked to the development of various non-communicable diseases (NCDs) [5,6], such as type II diabetes and cancer [5,7], and is therefore a major issue for public health and economic development worldwide [8].

Various interventions have been launched to reduce the risk of obesity and other NCDs. In Italy, the national guidelines for the prevention and treatment of obesity [9] emphasize strategies that encourage individual behavior change, particularly focusing on dietary habits and physical activity. Among these recommendations, special emphasis is placed on adopting the Mediterranean diet [10,11]. This dietary pattern has been shown to reduce mortality and the occurrence of NCDs [9,12,13], contributing to the reduction of body fat and the prevention of obesity [14].

One approach frequently tested to encourage behavioral change in the general population is digital dietary interventions [15]. These tools offer easily accessible methods to support health education and promote positive lifestyle changes [16]. Several studies reviewed by Benajiba et al. (2021) [17] confirm the effectiveness of digital dietary interventions in promoting adherence to the Mediterranean diet and improving dietary habits. However, researchers have also noted significant variability in their efficacy, indicating that many design-related factors in digital interventions still require thorough evaluation [18]. Overall, despite the growing body of scientific literature, findings on their effectiveness remain inconclusive.

The effectiveness of these interventions depends on multiple factors [19], among which user engagement and participant retention—commonly referred to as attrition—play a critical role [20]. Attrition refers to the phenomenon in which participants discontinue their involvement in an intervention or are lost to follow-up, thereby failing to receive the full benefit of the proposed program, regardless of its intrinsic efficacy. This issue poses a significant challenge to the validity and generalizability of intervention outcomes.

An increasing number of studies have sought to identify design features that minimize attrition and enhance participant engagement in digital health interventions [21]. Nevertheless, user engagement remains a complex and not fully understood issue.

Although the existing literature has predominantly examined how the design of digital interventions affects attrition rates and overall effectiveness [21], comparatively less attention has been paid to the individual-level determinants of engagement. Research in this area has mainly focused on demographic characteristics [22], especially considering age in association with digital literacy [18] and self-perceived efficacy [23].

Among the psychological factors that may influence an individual’s decision to commit to a digital dietary intervention is the importance assigned to the perceived health-related and hedonic characteristics of food in guiding dietary choices [24]. Conversely, craving sweets, using food as a reward, and perceiving food as a source of pleasure are negatively correlated with adherence to healthy dietary patterns. These traits are reliable predictors of overall eating habits [25] and are associated with consumer purchasing and cooking behavior [26]. Understanding the relationship between these behavioral traits and user engagement in digital dietary interventions can contribute to optimizing the design of digital tools and enhancing their overall effectiveness.

This study examined the effectiveness of the EATMED web application in improving adherence to the Mediterranean diet. This tool was designed to encourage the adoption of the Mediterranean diet by rewarding the purchase of food products aligned with its nutritional guidelines. A previous pilot study, conducted using a prototype of the application called *Yourpappa*, demonstrated promising results in terms of dietary behavior changes [27]. Building on these initial findings, the web application was comprehensively updated to improve both its interface and functional features. The main behavioral change technique used by this web app is the provision of rewards [28,29], which aims to promote food purchasing behaviors consistent with the Mediterranean diet and, consequently, to modify individual dietary habits [30,31]. Considering the association between food purchase and dietary profile [30,32], intervening in purchase decisions is a reliable strategy to improve the healthiness of the diet compatibly with the different dietary habits of a cohort of people, with positive results [33]. Multiple reviews have reported the positive influence of adopting a theory-based approach to encourage behavior change [18,19,27], emphasizing the importance of study design in effectively achieving intervention goals. These techniques can be more effectively implemented by understanding some of the most common drivers of action and inaction, ultimately leading to a more customized approach, recognized as a critical success factor in digital health interventions [19].

Given the relationship between impulsivity, time discounting, and diet-related NCDs [34], the Health and Taste Attitude Scale (HTAS) was included as a psychometric measure in the delayed reward intervention, as individuals with certain HTAS profiles may be more susceptible to developing overweight and obesity [26].

Overall, the objectives of the present work are twofold: (1) to analyze the effects of individuals’ demographic and behavioral characteristics—particularly health and taste attitudes—on engagement and attrition in a digital dietary intervention; and (2) to evaluate the effectiveness of the EATMED web application in enhancing adherence to the Mediterranean diet within a workplace intervention setting.

## 2. Materials and Methods

### 2.1. Study Design

An intervention study was conducted, comparing the self-reported adherence to the Mediterranean diet and dietary pattern in two moments during six months, separately for two cohorts of people: one cohort (Test) accessing the EATMED web app and the other cohort, who had received only written guidelines for the Mediterranean diet (Control). All the study’s procedures were performed online. Participants were recruited for the Test and Control cohorts by distributing a survey to verify eligibility criteria.

Both cohorts received a brochure containing information about the Mediterranean diet and general dietary guidelines, while only the Test cohort also received credentials to access a dedicated account on the EATMED web app. After six months, both cohorts received another survey to estimate the effect of the intervention in the Test cohort in comparison with the Control cohort.

This study was approved by the Ethics Committee of the University of Gastronomic Sciences of Pollenzo, Italy (Ethics Committee proceedings n. 10,012,024 and following amendment of 18 December 2024), and carried out according to the Declaration of Helsinki.

### 2.2. Participants

The participants for the Test cohort (N = 62, 59.7% female) were recruited by distributing an eligibility survey through the internal communication system of two Italian companies (CNH and Andriani S.P.A.), who collaborated to the study through their employee network. The survey verified the inclusion criteria to participate in the intervention: the absence of food allergies, a BMI between 18.5 and 34.9, and an age between 18 and 67 years, in order to include people in working age and to exclude people with obesity of class 2 and 3 and underweight. According to a power analysis based on the result of the previous study [27], to estimate a change in MEDI-lite score as the one reported in the previous analysis with a statistical power above 0.9, the minimum amount of participants required was 57; therefore, all 62 subjects recruited from this stage were included in the Test cohort.

Due to the limited availability of suitable individuals within the company, participants for the control group were recruited from the general population. Particular care was taken to ensure the absence of significant differences in general characteristics between the two groups by applying the same selection procedure: the same survey used for the Test cohort was distributed to recruit participants for the Control cohort (N = 92, 61.9% female), using social media accounts of the University of Gastronomic Sciences of Pollenzo on LinkedIn, and the newsletters dedicated to the partners of the University.

All participants provided their informed consent before answering the surveys and joining the intervention study.

### 2.3. Online Surveys

All surveys were created using Qualtrics XM© Software (2025 Qualtrics). All surveys were collected online by distributing a link to the first eligibility survey through the internal mailing service of two Italian private companies that collaborated in recruiting participants among their employees. Ineligible participants received an automatic message at the end of the survey, while eligible participants were invited to continue the experiment by providing their email address. Subsequently, the email was associated with an anonymous ID. To ensure anonymity, further communication was sent using an R script (version 4.4.2; [35]) that delivered messages to the email addresses without allowing the researcher to access participant data. All eligible participants who agreed to take part received a link to the second Qualtrics survey via automated email. This survey contained a MEDI-lite questionnaire [36] to estimate the adherence to the Mediterranean diet and the HTAS.

Respondents who completed the second survey received an invitation link to access the EATMED web app. After six months, a follow-up survey was sent to all participants containing the MEDI-lite questionnaire.

To associate answers from the same participant across different surveys while ensuring anonymity, each participant was assigned two random strings after the eligibility survey. These strings were automatically included in the email content along with the Qualtrics link, ensuring accurate matching between the anonymized ID and the string.

Each survey required participants to input the code included in the email as the first question, following the informed consent statement, allowing responses to be linked to the anonymized ID without exposing the email address.

For the Control cohort, an initial survey containing eligibility criteria, MEDI-lite, HTAS, and demographic questions was distributed using social media accounts (e.g., LinkedIn) and the newsletter dedicated to the business partners of the University of Gastronomic Sciences of Pollenzo. Eligible participants were asked for permission to provide their email address to receive the second part of the survey.

After six months, a second survey containing the MEDI-lite questionnaire was sent to Control cohort participants who had consented to continue, using an automated email containing the Qualtrics link. To match the first and second responses, another set of random strings was associated with each participant and automatically included in the email with the second link. On average, for the Test cohort the completion of eligibility survey for took 15 min, and the initial time survey took 10 min. The eligibility and initial time survey for the Control cohort took 20 min. The closing survey took 10 min for both the Test and Control cohorts.

#### 2.3.1. Demographic Questions

All participants from the Control and Test cohorts were asked to provide their age, gender (male, female, other, I prefer not to answer), height (in centimeters), weight (in kilograms), nationality, diet (omnivorous, flexitarian, vegetarian, vegan), and the context where they live (village/rural context (<10,000 inhabitants); town (10,000–70,000 inhabitants); city (>70,000 inhabitants)) [36].

#### 2.3.2. MEDI-Lite Questionnaire

The MEDI-lite questionnaire was organized into 9 different blocks [34], each corresponding to a food category: vegetables (two items: raw vegetables and cooked vegetables), fish (one item), legumes (one item), dairy (four items: milk, yogurt, fresh cheese, and seasoned cheese), fruits (two items: medium/large fruits and small fruits), extra-virgin olive oil (one item), cereals (five items: main course, bread, biscuits, breakfast cereals, and pizza), meat (three items: white meat, red meat, and cured meats and sausages), and alcohol (one item).

First, participants were asked about the consumption frequency of each food type and the usual portion size they consumed. The MEDI-lite score was then calculated according to the procedure reported by Sofi et al. [37]. A higher MEDI-lite score corresponds to a higher adherence to the Mediterranean diet.

#### 2.3.3. Health and Taste Attitude Scale

The Italian version of the HTAS questionnaire was presented, including six subscales [26], each composed of multiple items scored on a seven-point category scale (1 = disagree strongly; 7 = agree strongly). Each subscale contained a balanced number of negative statements to avoid systematic errors. Negative statements were reversed and re-coded for the calculation of the final scores. Three subscales were related to attitudes toward perceived health: general health interest (eight items, estimating the respondent’s interest in healthy eating); light product interest (six items, focusing on the intention to eat reduced-fat or reduced-sugar food products); natural product interest (six items, focusing on the intention to eat food that does not contain additives or is unprocessed).

For estimating attitudes toward food taste, three subscales were used: craving for sweets (six items, asking respondents to report the strength of cravings for chocolate, sweets, and ice cream); using food as a reward (six items, measuring attitudes toward using foods as a reward); pleasure (six items, relating to the importance of obtaining pleasure from food). After recoding negatively worded items, a mean score was computed from the individual scores for each participant and subscale.

### 2.4. The EATMED Web Application

A web-based digital application was designed to provide users with a platform for creating an individual account and interacting with the digital system. Once the account was activated, users could connect using a PC, laptop, or smartphone with an internet connection and an installed browser.

The preliminary design of EATMED was developed in a previous study [27], where the efficacy of the beta version (called Yourpappa) was tested in a cohort of users, reporting a higher increase in the MEDI-lite score ratings in the Test cohort compared to the Control cohort. In comparison with the previous research version, the web app in this study was largely revised and improved, leading to a new software tool developed considering the results obtained from the previous research.

The objective of the EATMED web app is to encourage adherence to the Mediterranean diet by rewarding healthy food purchase choices (i.e., the purchase of food types compatible with the Mediterranean diet in adequate amounts) through the assignment of a certain number of points that can be accumulated and redeemed for prizes. The mechanism for assigning rewards for healthy food purchases consists of granting points to users when they willingly report their purchase choices using their user accounts.

To report their purchase choices, users can access a dedicated web app section designed to upload an image of a shopping receipt. After the upload, Optical Character Recognition software embedded in the application backend proceeds to extract all the text strings from the image. Following the text extraction, a dedicated algorithm filters the strings, retaining only those that describe purchased items. Lastly, a string-matching algorithm synchronized with an internal database assigns each purchased item to a food category, which corresponds to a certain number of points according to the importance of that food category within the Mediterranean diet. The food categories used in the web app are reported in Table 1. Points are assigned based on the number of purchased items linked to each category, with a maximum number of times they can be registered weekly. When an item in a given category is uploaded more than the allowed maximum, it is still recorded in the database, but no points are awarded. This mechanism is intended to reward balanced consumption, discourage overconsumption, better reflect choices representative of the Mediterranean diet [38], and ensure that the maximum weekly amount of points could be obtained also by individual users who provide for small households.

The categories were defined according to the dietary recommendations for the Mediterranean diet as reported in [11]. Therefore, the highest number of points and the highest weekly upload limit are assigned to the food category vegetables, while the lowest number of points and upload limits are assigned to meat. The food category other food was created to include products not properly associated with the Mediterranean diet, such as processed meat, fruit juices, alcoholic beverages, soft drinks, nervine beverages, fresh and dry desserts, and packaged snacks.

As stated in [39], providing rewards for behavior is one of the main behavioral change techniques to encourage the adoption of a healthier dietary pattern [28]. To promote healthier choices, users were encouraged to increase the consumption of foods belonging to the categories in order to increase the number of weekly points they could obtain.

Besides the core feature, the user interface includes a main page showing the user an automatically updated dashboard displaying two barplots reporting the points collected from uploading their purchase choices and the estimated carbon footprint associated with the purchase items, estimated from the SUETABLElife dataset [39]. The barplots also report the average values for all the users to facilitate social comparison [28].

Furthermore, another section displayed the available prizes, allowing users to check whether the points collected were sufficient to request them.

In addition, every Monday, each user received a newsletter curated by the research team, containing motivational content about the Mediterranean diet: a healthy recipe with seasonal ingredients, general recommendations for a healthy diet, and a recap of the points collected by the user. Part of the content of the newsletter was created using GenAI (ChatGPT version GPT-4), under supervision of the research team. Lastly, users had access to an internal blog section containing informative material on various topics related to the Mediterranean diet.

The EATMED web app was developed using the PHP programming language, adopting the Laravel Framework for web development. The development of the web app has been done in collaboration with B4web s.r.l. IT Company.

### 2.5. Data Analysis

Power analysis was performed using Monte Carlo Simulation (n = 1000) [40] estimating the statistical power for a non-parametric paired Wilcox test using the mean and standard deviation for the starting population and the expected change in the MEDI-lite using the data from the previous study [27] in a range from 5 to 150 participants.

Demographic data between total Test cohort and total Control cohort were compared using chi-square test for discrete values and Wilcox test for continuous variables.

To estimate the factors affecting attrition in starting the intervention, a Generalized Linear Model (GLM) with a logistic link function was estimated using the participation in the intervention of each user as the response variable. The participation was coded as a binary variable by assigning 1 to the participants who used the EATMED web app (Active Users) and 0 to those who did not (Inactive Users), according to the attrition analysis proposed by [20]. To select the factors included in the final model, a selection procedure was performed using a recursive algorithm to optimize the Akaike Information Criterion by adding variables and first-level interactions to the null model to estimate the best-fit model, selecting the factors that are related to the variance contained in the variable [41]. The total of the factors tested consists of all the first-level interactions of the data collected from the MEDI-lite questionnaire, HTAS, and demographic questions collected from the Control cohort.

To estimate quantitatively participation and dropout for every active participant in the Test cohort during the intervention, the days between the web app launch and the date of the last receipt loaded in the system were counted. Subsequently, the effect of HTAS scores and socio-demographic characteristics on dropout during the trial period was estimated using a Cox survival model comparing the days counted with the total duration of the intervention [20]. To avoid overfitting, the model considered only the demographic characteristics of gender, age, BMI, and the six items from the HTAS questionnaire.

The effect of the intervention on overall dietary behavior was estimated by calculating a Linear Mixed Model (LMM), considering the effect on the MEDI-lite score at two time points (T0–beginning and T1–end, after six months), the two cohorts (Test and Control), and their interaction, including individuals as random factors. Subsequently, a post hoc pairwise test was applied for the interaction factor between time and cohort, adopting a least-squares marginal mean computation.

The significance level (α) adopted for all tests was 0.05. All analyses were conducted with R software (version 4.4.2 [35]). The GLM model estimation and optimization were estimated using the package “stats” [35], the Cox survival model was estimated using the package “survival” [42], the LMM was estimated using the package “lme4” [43], and the post hoc test was estimated with the package “emmeans” [44].

## 3. Results

### 3.1. Participants Data

Of the 184 participants who responded to the initial eligibility survey for the test cohort, 77 (41.8%) met the eligibility criteria. On the contrary, three participants (1.6%) were excluded due to specific dietary restrictions and food allergies, and one hundred and seven participants (58.1%) were excluded because they did not finish the eligibility survey.

Among the 77 eligible respondents of the test cohort, 62 (80.5%) finished the survey related to T0, while 15 people (19.5%) did not complete it. An EATMED account was created for each of the 62 participants and automatically associated with each of the respondents’ email addresses. For the control cohort, the Qualtrics survey collected 125 answers, of which 92 were considered valid. Contrarily, 23 participants (18.4%) were not included because they were not eligible according to the conditions of the study, and 10 participants were excluded due to incomplete answers (8.0%). According to a chi-square tests and Wilcox test, no significant differences were detected between the Control cohort and the total Test cohort. The demographic characteristics of the Test and Control cohort participants are reported in Table 2.

### 3.2. Attrition Analysis

During the intervention, a total of 757 receipts were loaded by the users in the EATMED system, with a total of 5386 food purchases. On average, each user loaded 32 receipts, with a minimum of 1 and a maximum of 123. The users used the EATMED web app on an average of 53 days, with a minimum of 1 day and a maximum of 213 days. Within all the test cohort, seven users kept using the web app until the end of the intervention.

Table 3 reports the coefficients and the significance of the best-fit model used to determine the active participation in the intervention. Moreover, the table shows the effect of the factors that were selected after performing a stepwise forward analysis, executed by adding progressively new factors to a blank model to estimate the optimal fit for the GLM with a logit link function. The reported results indicate the factors that determine the variation in the distribution of the incidence of attrition.

Results showed that the trait food as reward represents the strongest predictor for withdrawal from the Test in the early stage, while higher values of the MEDI-lite score and age represent factors encouraging slight adherence to the intervention. This model indicates that participants having an attitude toward considering food as a reward were less likely to participate in the intervention, and participants with a higher adherence to the Mediterranean diet were more likely to participate instead.

In Table 4 the coefficients and the significance of the Cox model determining the effects of HTAS and demographic parameters on the higher probability of dropout are reported.

Results showed that males with higher BMI scores and an attitude of seeking rewards from eating food were more likely to terminate the intervention prematurely. The estimated model confirmed how the attitude of food as reward remains a determinant of the engagement, indicating a consistent association with attrition. These results suggest a relationship with the engagement in dietary intervention studies and the attention to the use of sensory properties of food as a coping mechanism.

### 3.3. EATMED’s Effect on Adherence to the MD

**Figure 1:**
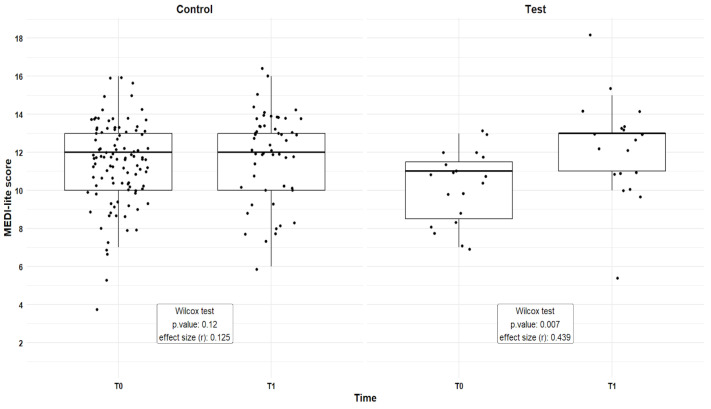
Boxplot reporting the MEDI-lite scores at the beginning (T0) and the end (T1) of the intervention study, both for the Control and Test cohorts. Each point represents an individual measurement, the thick line indicates the median value, and the box indicates the interquartile range.

The overall results of the MEDI-lite scores are reported in Figure 1, while, to estimate the effect of the intervention, Table 5 reports the result of the LMM of the effect of time and cohort on the MEDI-lite score, including the individual participants as random factors.

The Control cohort had at the beginning and the end of the intervention a MEDI-lite score median value of 11.4 and 11.8, respectively, while the Test cohort moved from an initial mean score of 10.2 to a final mean of 12.2, with a significant difference confirmed by the Wilcox test, with an effect size index r of 0.439.

The LMM analysis showed that there is a significant difference in the MEDI-lite score between T0 and T1 within the two cohorts, as we can see by checking the interaction between the factors time and cohort.

In Table 6 the results of the post hoc analysis highlighted a significant difference between the Control and Test cohorts at initial time and final time, and a significant improvement of the Test cohort after the intervention in comparison to the initial results.

Comparing the results, time itself has not changed the adherence to the Mediterranean diet, but there is strong evidence that the test cohort reported a different adherence rate to MD after the intervention, while the Control cohort remained on the same median values.

## 4. Discussion

As reported in multiple reviews [15,17,19,21], there is a growing body of literature about digital dietary interventions. Studies that propose tools similar to EATMED include e-12HR [22] and the Credits4Health project [45], although our research provided new information on both the effect of attitudes on commitment to digital intervention and the efficacy of a digital nutrition intervention based on rewards in promoting positive eating behavior.

The attrition rate of our study (61.8%), although significant, is comparable to the attrition rates reported in other digital interventions aimed at improving adherence to the MD and healthy eating [21,46,47,48], which reported dropout rates between 60% and 70% across multiple interventions. Those studies did not report specific information about the socio-demographic characteristics of the dropouts, but from the data collected, a relationship between perceived self-efficacy and engagement has been suggested [21].

The attitude of perceiving food as a reward significantly influenced both initial engagement and sustained participation in the intervention. This finding suggests a potential conflict between the delayed reward offered by the intervention and participants’ inclination toward immediate gratification through food. Previous research has highlighted how seeking immediate rewards from food is associated with a higher incidence of unhealthy diets [21], and other studies have reported an association between greater self-regulation capacity and adherence to a healthy dietary pattern [49]. Similarly, a stronger attitude toward using food as a reward is associated with individuals more attracted to unhealthy diets and less interested in adhering to a healthy diet [26,50], with a higher preference for high-fat food [51].

The results of our study highlighted the role of attitude toward food taste as a discouraging factor in dietary intervention, showing that the online administration of dietary recommendations is still influenced by the attitudes of participants. While our research findings showed that a stronger attitude toward taste was associated with a higher dropout rate, unfortunately, a stronger attitude toward health did not correspond to a significantly lower dropout rate. The items related to health, such as general health interest, light product interest, and natural product interest, did not affect engagement with the dietary intervention, either positively or negatively. These results align with the observations from [26], reporting that a stronger attitude toward health corresponded to a higher likelihood of following a healthy diet, but not to increased attention to product information or convenience in meal preparation.

Overall, the intervention showed a significant effect of the use of the EATMED web app on the MEDI-lite score of participants, suggesting that participants who did not drop out of the intervention improved their dietary habits. In our study, participants belonging to the Test cohort increased their adherence to the Mediterranean diet by two points, according to the MEDI-lite score, while the Control cohort increased by only four-tenths of a point. In this context, it is interesting to note that a study conducted on 208 patients who attended the Clinical Nutrition Unit of Careggi University Hospital, Florence, demonstrated that the MEDI-lite score provided significant protection against the risk of abdominal obesity for every one-unit increase in the total score (OR 0.72, 95% CI 0.63–0.82; *p* < 0.001) [52]. In another study, researchers investigated the association between adherence to the Mediterranean diet (through the MEDI-lite score) and the likelihood of being obese in 1814 Italian individuals [53]. The authors found an inverse correlation between the two. The data available in the literature, combined with the results from our web app, allow us to hypothesize that EATMED could contribute to reducing the risk of obesity as part of a broader strategy of tools aimed at combating overnutrition.

In a study using data from the Concord Health and Ageing in Men Project (CHAMP), the authors analyzed the connection between circulating cytokine levels, musculoskeletal health, incident falls, and adherence to the Mediterranean diet using the MEDI-lite score. Data were collected from 616 elderly participants with a mean age of 81.1 ± 4.5 years. Results showed that higher adherence to the Mediterranean diet was associated with higher appendicular lean mass adjusted for body mass index (ALMBMI) (β: 0.004 kg; 95% CI: 0.000, 0.008) and lower incident fall rates (IRR: 0.94; 95% CI: 0.89, 0.99) [54]. As highlighted by a recent review of the literature, in the coming years we will witness a doubling of the elderly population who, very often, spend the last years of their lives with one or more disabilities. It is therefore necessary to promote healthy aging, and the Mediterranean diet represents an excellent tool [55]. Therefore, we believe that new tools, such as EATMED, should be evaluated for their potential to promote healthy aging.

Finally, from a One Health approach perspective, the literature suggests that the Mediterranean diet is both a healthy and sustainable diet [10]. In particular, in a recent study [56], adherence to the Mediterranean diet was evaluated using the MEDI-lite score in 29,210 French volunteers. The researchers showed that a higher MEDI-lite score was associated with a reduced environmental impact of the dietary pattern. In our web app, we encouraged and promoted the Mediterranean diet as a healthy and sustainable model. Indeed, users had personal graphs available to evaluate the CO_2_ impact of their food choices. All these findings indicate that using purchasing choices as a leverage point to encourage dietary change is effective in terms of the One Health concept. Nevertheless, the attrition rate suggests that the digital tool could still benefit from further improvements.

Although the results offer insights into digital health interventions, it is important to acknowledge the limitations of this study. First, the recruitment procedure for the Test cohort was focused on enlisting participants from a specific workplace, limiting the ability to reach a broader population that might be interested in or benefit from such a dietary intervention. While these results reflect a real-life intervention scenario, the difference in recruitment procedures between the Control and Test cohorts resulted in differences in initial adherence to the MD score and an unbalanced number of participants across cohorts. Another issue that is important to acknowledge is the effect of the dropout during the app usage, which caused discontinuous use of the tool by a fraction of the users. Unfortunately, those factors reduce the statistical power of the results, undermining the possibility of generalizing these findings to the broader population.

Furthermore, comparing the features of the EATMED web app with the factors identified in the literature [19] as contributing to the success of digital interventions, it appears that although the EATMED web app includes personalized features, such as dashboard-reported data, it does not provide individualized feedback for each purchase choice—representing a viable area for improvement, considering the positive association reported between adherence to the Mediterranean diet and nutrition knowledge [57].

Although the positive effect of the intervention was demonstrated, the results could vary in cohorts with different attitudes, socio-demographic characteristics, and food cultures.

## 5. Conclusions

The digital intervention conducted through the EATMED web application demonstrated a multifaced impact on participants’ behavior. Notably, the study highlighted that the individual attitude toward health and taste significantly plays a significant role in both the initial engagement and sustained participation in a dietary intervention. These findings underscore the importance for health and nutrition professionals to consider such motivational factors when designing effective strategies for promoting healthier lifestyles. In particular, tailoring or adapting interventions for individuals at higher risk of dropout—who often coincide with those most vulnerable to diet-related NCDs—may enhance the overall effectiveness and retention of such programs. Addressing attitudinal differences could therefore be a key element in encouraging both willingness and effective participation in dietary intervention, ultimately contributing to reach the necessary public health goals. Furthermore, the observed short-term increase of adherence to the Mediterranean diet within the Test cohort indicates the potential effectiveness of the EATMED digital tool. These promising results encourage the need for rather evaluation and refinement of the tool, with the goal of scaling its implementation and assessing its long-term impact on dietary patterns and health outcomes.

## Figures and Tables

**Table 1:** Food categories comprised in the EATMED web app.

Food Category	Points per Unit	Weekly Uploading Limit
Other foods	0	100
MEAT (*red meat, poultry*)	5	1
CEREALS (*bread, pasta, rice, rusk, flour, etc*.)	15	2
FRUIT (*fresh fruit, fruit in syrup, canned fruit,* etc.).	15	3
DRIED FRUIT (*walnuts, hazelnuts, almonds, pistachios, cashews, peanuts,* etc.).	15	2
Dairy products (*milk, yogurt, and kefir*)	10	2
CHEESE and BUTTER	10	1
LEGUMES (*peas, chickpeas, lentils, beans, broad beans, soybeans, etc*.)	15	2
EXTRA-VIRGIN OLIVE OIL	15	1 every two weeks
FISH (*fresh fish, frozen fish, canned fish, etc*.)	10	2
VEGETABLES (*fresh, frozen vegetables, minestrone, tomato puree, creamed vegetables, etc*.)	20	3
EGGS	10	1

**Table 2:** Demographic characteristics of the Test and Control cohort participants.

Socio-Demographic Characteristics	Levels	Test Cohort			Control Cohort
		Total	Active	Not Active	Total
Gender (*p*-value Test vs. Control: 0.0916)	Female	37 (59.7%)	13 (54.2%)	24 (63.2%)	57 (62.0%)
	Male	25 (40.3%)	11 (45.8%)	14 (36.8%)	35 (38.0%)
BMI * (*p*-value Test vs. Control: 0.4039)		22.9 ± 2.9	22.9 ± 2.9	23.2 ± 2.8	23.0 ± 3.8
Age * (*p*-value Test vs. Control: 0.3273)		41.9 ± 8.1	43.2 ± 7.6	41.1 ± 7.6	42.8 ± 15.1
Nationality (*p*-value Test vs. Control: 1)	Italian	57 (91.9%)	22 (91.7%)	35 (92.1%)	90 (97.8%)
	Non-Italian	5 (8.1%)	2 (8.3%)	3 (7.9%)	2 (2.2%)
Diet (*p*-value Test vs. Control: 0.2381)	Omnivore	50 (80.6%)	17 (70.8%)	33 (86.8%)	71 (77.2%)
	Flexitarian	8 (12.9%)	3 (12.5%)	5 (13.2%)	16 (17.4%)
	Vegetarian	2 (3.2%)	2 (8.3%)	0 (0%)	4 (4.3%)
	Vegan	2 (3.2%)	2 (8.3%)	0 (0%)	1 (1.1%)
Social Context (*p*-value Test vs. Control: 0.1991)	Big town (*More than 70,000 inhabitants*)	28 (45.2%)	9 (37.5%)	19 (50.0%)	43 (46.7%)
	Medium town (*More than 10,000 and less than 70,000 inhabitants*)	19 (30.6%)	7 (29.2%)	12 (31.6%)	35 (38.0%)
	Small town (*Less than 10,000 inhabitants*)	15 (24.2%)	8 (33.3%)	7 (18.4%)	14 (15.3%)
	Total	62	24 (38.7%)	38 (61.3%)	88

For each socio-demographic level, the count and the relative occurrence are reported in brackets, except for the variable labeled with *, for which mean ± standard deviation have been reported. *p*-value reported in brackets below each socio-demographic characteristic indicates the differences are for the chi-square test comparing the percentages of occurrence in the total test cohort and in the total control cohort, except for variables labeled with *, which is estimated from a Wilcox test.

**Table 3:** Results of the final model estimated using stepwise optimization of the GLM model predicting the incidence of attrition.

Coefficient	Estimate	Std. Error	Z Value	*p*-Value
(Intercept)	1.43	3.14	0.455	0.6492
HTAS food as reward	−1.06	0.36	−2.984	0.0028 **
Age	0.07	0.04	1.728	0.0840 #
MEDI-lite score	0.32	0.16	1.973	0.0485 *
HTAS general health interest	−0.72	0.44	−1.625	0.1042

# = *p*-values between 0.1 and 0.05, * = *p*-values between 0.05 and 0.01, ** = *p*-values between 0.01 and 0.001.

**Table 4:** Results of the Cox survival model estimated on the days of use before dropout.

Characteristic	HR	95% CI	*p*-Value
Gender			
Female	—	—	
**Male**	**0.15**	**0.03**–**0.68**	0.0144 *
Age	0.93	0.85–1.03	0.1553
**BMI**	**1.31**	**1.01**–**1.69**	0.0206 *
HTAS			
Craving for sweets	0.67	0.37–1.21	0.1178
**Food as a reward**	**0.3**	**0.11**–**0.76**	0.0169 *
General health interest	0.37	0.11–1.26	0.0688 #
Pleasure	1.96	0.85–4.52	0.1637
Light product interest	1.03	0.67–1.59	0.9679
Natural product interest	1.89	0.93–3.83	0.0755 #

CI = Confidence Interval, HR = Hazard Ratio, # = *p*-values between 0.1 and 0.05, * = *p*-values between 0.05 and 0.01. Bold format indicates *p*-value below 0.05.

**Table 5:** Results of the LMM to estimate the effect of the factors of the intervention study factors on the MEDI-lite score.

Factor	F	Df	Df.res	*p*-Value
**(Intercept)**	**2666.79**	**1**	**200.56**	<0.0001 ***
Time	1.02	1	93.12	0.3147
**Cohort**	**29.50**	**1**	**201.21**	<0.0001 ***
Time:Cohort	13.95	1	101.76	0.0003

*** = *p*-values below 0.001. Bold format indicates *p*-value below 0.05.

**Table 6:** Results of the least-squares estimated test for the post hoc analysis of the interaction factor time/cohort.

Comparison	Estimate	SE	Df	t. Ratio	*p*-Value
T0 Control–T1 Control	−0.29	0.28	93.12	−1.01	0.7434
**T0 Control–T0 Test**	**1.96**	**0.36**	**201.21**	**5.43**	>0.0001 ***
T0 Control–T1 Test	−0.34	0.51	219.09	−0.67	0.9101
**T1 Control–T0 Test**	**2.24**	**0.40**	**228.88**	**5.54**	>0.0001 ***
T1 Control–T1 Test	−0.06	0.55	210.86	−0.10	0.9996
**T0 Test–T1 Test**	**−** **2.30**	**0.46**	**105.28**	−**5.01**	>0.0001 ***

*** = *p*-values below 0.001. Bold format indicates *p*-value below 0.05.

## Data Availability

The original data presented in the study in anonymized are openly available in OSF.io at https://osf.io/sda73/?view_only=ca4d1d60e22644c48f487d0cd19aab89 (accessed on 1 May 2025) or “DMD–DIGITAL MEDITERRANEAN DIET–Use of a digital platform to promote the Mediterranean diet [DMD–DIGITAL MEDITERRANEAN DIET–Utilizzo di una piattaforma digitale per la promozione della dieta mediterranea]”.

## Abbreviations

The following abbreviations are used in this manuscript:

GLM	Generalized Linear Model
HTAS	Health and Taste Attitude Scale
LMM	Linear Mixed Model
MD	Mediterranean Diet
NCD	Non-Communicable Diseases
